# Genomic characterization of *Sinorhizobium meliloti* AK21, a wild isolate from the Aral Sea Region

**DOI:** 10.1186/s40064-015-1062-z

**Published:** 2015-06-16

**Authors:** María Dolores Molina-Sánchez, José Antonio López-Contreras, Nicolás Toro, Manuel Fernández-López

**Affiliations:** Grupo de Ecología Genética, Departamento de Microbiología del Suelo y Sistemas Simbióticos, Estación Experimental del Zaidín, CSIC, Calle Profesor Albareda 1, 18008 Granada, Spain

**Keywords:** Comparative genome hybridization, Suppression subtractive hybridization, Sm14kOligo microarrays, *Sinorhizobium meliloti*, Phenotypic microarrays, Nitrogen fixation

## Abstract

The symbiotic, nitrogen-fixing bacterium *Sinorhizobium meliloti* has been widely studied due to its ability to improve crop yields through direct interactions with leguminous plants. *S. meliloti* AK21 is a wild type strain that forms nodules on *Medicago* plants in saline and drought conditions in the Aral Sea Region. The aim of this work was to establish the genetic similarities and differences between *S. meliloti* AK21 and the reference strain *S. meliloti* 1021. Comparative genome hybridization with the model reference strain *S. meliloti* 1021 yielded 365 variable genes, grouped into 11 regions in the three main replicons in *S. meliloti* AK21. The most extensive regions of variability were found in the symbiotic plasmid pSymA, which also contained the largest number of orthologous and polymorphic sequences identified by suppression subtractive hybridization. This procedure identified a large number of divergent sequences and others without homology in the databases, the further investigation of which could provide new insight into the alternative metabolic pathways present in *S. meliloti* AK21. We identified a plasmid replication module from the *rep*ABC replicon family, together with plasmid mobilization-related genes (*tra*G and a VirB9-like protein), which suggest that this indigenous isolate harbors an accessory plasmid. Furthermore, the transcriptomic profiles reflected differences in gene content and regulation between *S. meliloti* AK21 and *S. meliloti* 1021 (ExpR and PhoB regulons), but provided evidence for an as yet unknown, alternative mechanism involving activation of the *cbb*3 terminal oxidase. Finally, phenotypic microarrays characterization revealed a greater versatility of substrate use and chemical degradation than for *S. meliloti* 1021.

**Electronic supplementary material:**

The online version of this article (doi:10.1186/s40064-015-1062-z) contains supplementary material, which is available to authorized users.

## Background

Since its discovery in 1888, the biological fixation of N_2_ (BFN) has been considered a potentially useful tool for land remediation (Peoples et al. 1995; Hellriegel and Wilfarth 1888). The symbiotic interaction between the soil bacterium *Sinorhizobium meliloti* and the leguminous plant *Medicago sativa* is one of the most widely studied models. *S. meliloti* is found in many different soil environments around the world, suggesting that this species has broad metabolic adaptation capabilities (Souza et al. 1992; Paffetti et al. 1996; Jebara et al. 2001; Roumiantseva et al. 2002; Biondi et al. 2003; Giuntini et al. 2005; Bailly et al. 2007; Jones et al. 2007; Stiens et al. 2008; Schneiker-Bekel et al. 2011). Investigations of genome dynamics have been carried out at the intra- and interspecies levels in recent years in intensive studies of natural isolates of related rhizobia, discriminating between core and accessory genes (Guo et al. 2009; Galardini et al. 2011, 2013a, b; Bailly et al. 2011; Epstein et al. 2012; Sugawara et al. 2013). The complete annotated genome sequence of *S. meliloti* isolated from diverse soil types (agricultural fields, contaminated soils,…) and continents (1021, SM11, BL225C, AK83, GR4, Rm41) can now be obtained from databases (Galibert et al. 2001; Schneiker-Bekel et al. 2011; Galardini et al. 2011; Martínez-Abarca et al. 2013), and several other draft genomes are also available (Galardini et al. 2013a, b).

Fixation rates in saline environments are highly dependent on the physiological state of the host plant and the salt tolerance of the rhizobia. Salt stress decreases the nodulation of leguminous plants, by inhibiting very early symbiotic events (Zahran 1991). Salinity therefore has a major impact on soil usage in agriculture. Remarkably, about 40% of soils worldwide have high salt contents and are generally considered to be unproductive. The general solution applied to overcome this problem in recent decades has been the extensive use of chemical fertilizers and salt-tolerant plants (Zahran 1999). However, the production and application of fertilizers are costly practices, both economically and ecologically. This led to efforts to isolate salt-tolerant plants and rhizobial strains mediating efficient nodulation in saline conditions, which have met with limited success (Ibragimova et al. 2006; Moawad and Beck 1991; Craig et al. 1991; Mohammad et al. 1991; Chien et al. 1992; Zou et al. 1995; El-Sheikh and Wood 1995; Lal and Khanna 1995; Mashhady et al. 1998; Hashem et al. 1998; Ohwada et al. 1998). In recent years, the emphasis has shifted to the description of genes conferring salt tolerance in free-living rhizobia and in rhizobia living in symbiosis with plants (Nogales et al. 2002; Kanesaki et al. 2002; Wei et al. 2004; Han et al. 2005; Domínguez-Ferreras et al. 2006, 2009). For instance, the trehalose biosynthesis has a role in *S. meliloti* osmotolerance and nodule occupancy (Domínguez-Ferreras et al. 2009) and *noe*J, involved in LPS biosynthesis, seems to indirectly control salt tolerance in *R. tropici* (Nogales et al. 2002). *S. meliloti* AK21 has been isolated from nodules of *Medicago sativa* L. subsp. ambigua growing in the North Aral Sea Region, an area severely hit by drought, salinity and the effects of pollution (Ibragimova et al. 2006). Here, we aim to establish the main features of *S. meliloti* AK21 in comparison to the reference strain *S. meliloti* 1021. Genomic similarities were defined by comparative genome hybridization (CGH) (Salama et al. 2000; Murray et al. 2001; Malloff et al. 2001; Cummings et al. 2004; Rajashekara et al. 2004; Giuntini et al. 2005; Taboada et al. 2007; Wu et al. 2008; Guinane and Fitzgerald 2008; Carter et al. 2008), and the specific genetic differences were revealed by suppression subtractive hybridization (SSH) (Lukyanov et al. 1994; Diatchenko et al. 1996; Gurskaya et al. 1996; Akopyants et al. 1998; Galbraith et al. 2004). We also carried out transcriptional and phenotypic characterization of the natural isolate *S. meliloti* AK21 by microarray-based cDNA hybridization and with a phenotype microarray system (Biolog), respectively. Higher levels of sequence variability were observed in the megaplasmid pSymA than in the other replicons. Moreover, we found that *S. meliloti* AK21 also contained newly acquired genes, probably organized into an accessory plasmid, potentially accounting for the fitness of this wild isolate. Finally, *S. meliloti* AK21 displayed major differences in gene expression with respect to *S. meliloti* 1021, which may point toward the factors underlying nodulation efficiency in saline conditions.

## Results

### Comparative genomic hybridization reveals large missing regions in *S. meliloti* AK21 compared to *S. meliloti* 1021 genome

**Figure 1:**
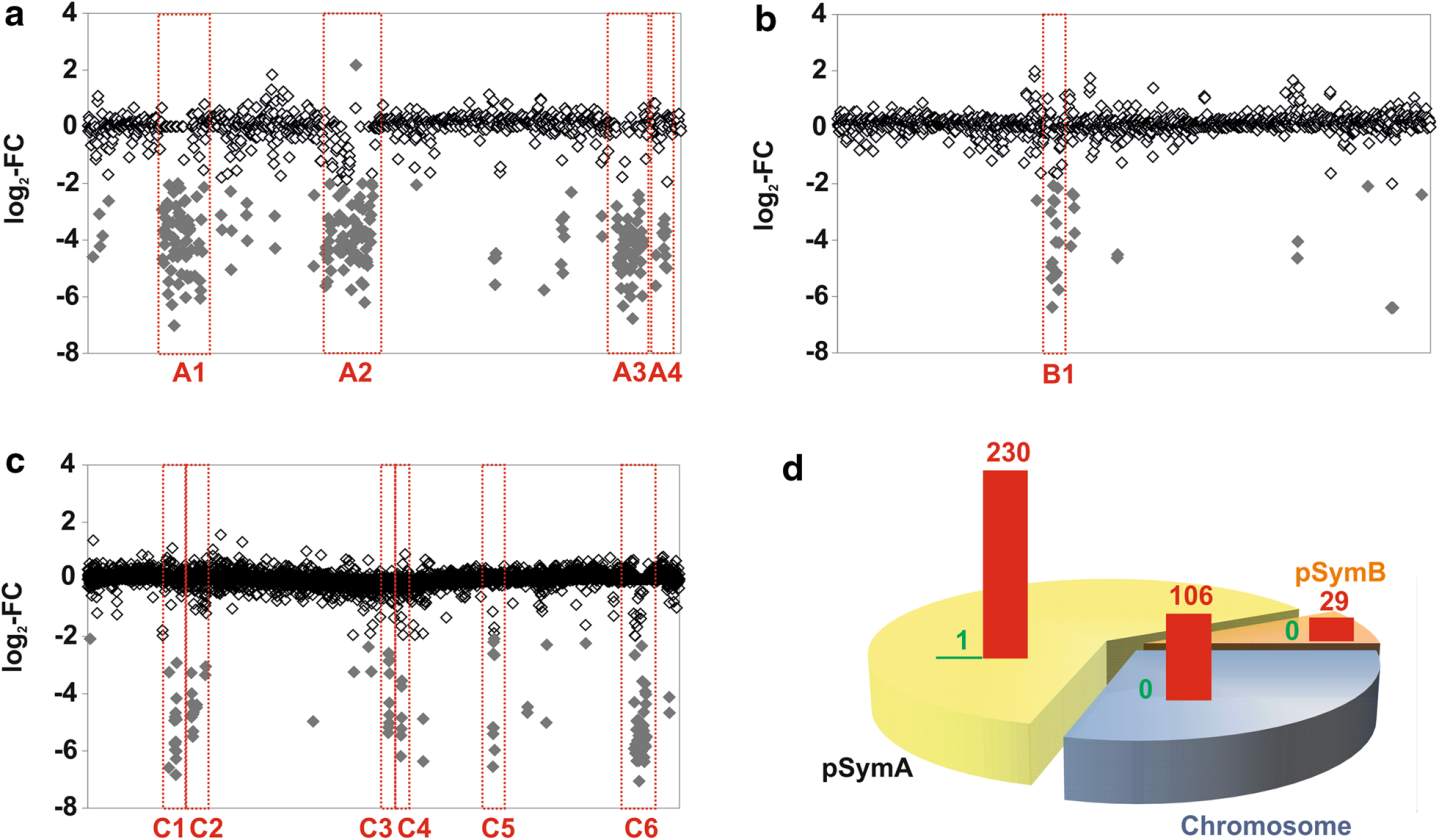
Genomic clusters missing from *S. meliloti* AK21, identified by comparative genome hybridization. Labeled total DNA from *S. meliloti* AK21 was competitively hybridized with labeled DNA isolated from *S. meliloti* 1021 on an Sm14kOligo microarray. Statistically significant signals (*p* value <0.05) are shown individually for the megaplasmid pSymA (**a**), chromid pSymB (**b**) and the chromosome (**c**), respectively. The *y*-axis shows the log_2_-fold change (M value), corresponding to the base 2 logarithm of the ratio of intensities between Rm1021 and *S. meliloti* AK21 intensity signals. Genes are represented along the *x*-axis, in the order of their NCBI-annotated location on the corresponding replicon. Genes with an M-value below -2 or greater than 2 are highlighted as *grey diamonds* and the missing gene clusters are boxed in *red*. **d** Diagram representing the distribution of variable genes between the three replicons: chromosome, pSymB chromid and pSymA megaplasmid. *Vertical bars* indicate the number of genes with a major number of copies (in *green*) and absent or highly divergent genes (in *red*).

Genomic differences between the salt-tolerant strain *S. meliloti* AK21 and the reference strain *S. meliloti* 1021 (hereafter referred to as Rm1021) were studied by comparative genome hybridization (CGH). The sequence of the *S. meliloti* AK21 genome has not yet been published, albeit Eckhardt lysis analysis revealed the presence of four replicons, three large ones probably corresponding to the chromosome, pSymA and pSymB, and a 120 kb-length plasmid (Ibragimova et al. 2006; M. Bazzicalupo personal communication). It is known that other sequenced strains of *S. meliloti* present a genomic organization similar to Rm1021 (Galardini et al. 2011) which was annotated with 6,292 genes distributed in the three replicons: 3,429 genes in the chromosome; 1,293 genes in pSymA; and 1,570 genes in pSymB (Galibert et al. 2001). We detected 365 protein-coding genes differing between the two strains (equivalent to 5.8% of the annotated Rm1021 genome, Additional file 1), most of which were absent from or markedly different in *S. meliloti* AK21, with only one of these genes displaying a lower signal intensity in Rm1021 (SMa1073, 89% identity to an ATP-binding protein). Missing regions were identified in *S. meliloti* AK21 which correspond to regions localized in the three major replicons of Rm1021 (Figure 1). In accordance with previous CGH analyses in *S. meliloti* (Giuntini et al. 2005; Stiens et al. 2008), most of the genome variations in *S. meliloti* AK21 (230 genes) would be located on megaplasmid pSymA (Figure 1a). By contrast, pSymB emerged as the most stable replicon, with only minor variations (29 genes; Figure 1b), consistent with the widespread view that this plasmid behaves like a true chromosome (Finan et al. 2001; Schneiker-Bekel et al. 2011; diCenzo et al. 2013; Galardini et al. 2013b). Finally, the chromosome displayed moderate variability in *S. meliloti* AK21, with 106 genes identified as variable (Figure 1c). All variable genes were annotated using Blast2GO and the NCBI database (Additional file 1). Genes encoding transposases were the most abundant, accounting for 23.6% of the deleted/mutated genes in *S. meliloti* AK21, followed by hypothetical/unknown genes (7.82%), transcriptional regulator genes (6.68%) and genes encoding ABC membrane transporters (5.45%). As expected, the minor variable genes were related to general metabolism (3.06%).

Further analysis of the raw data revealed that *S. meliloti* AK21 lacked 11 genomic clusters present in Rm1021 (Figure 1). These missing gene clusters are described in detail in Table 1. Remarkably, most of these gene clusters are flanked either by sequences with a highly complex secondary structure (generally corresponding to small RNAs) or transposase/insertion sequences (IS). Moreover, those located on the chromosome generally preceded tRNA-encoding sequences. They may therefore be considered potential “hot spots” for horizontal gene transfer, due to the presence of large numbers of IS and other external origin elements (such as putative phage-encoded protein sequences). Six of these missing regions had already been identified as absent from the chromosome [I (C1); II (C2); III (C6)] and pSymA megaplasmid [II (A2); IV (A3); V (A4)] in *S. meliloti* SM11 (Stiens et al. 2008). The other five regions were located on the chromosome (3), the chromid pSymB (1), and the megaplasmid pSymA (1). The missing chromosome clusters corresponded to regions mostly containing genes encoding conserved hypothetical proteins and transporters, or metabolic enzymes (Table 1; Additional file 1). The B1 region encodes several proteins involved in capsular polysaccharide synthesis and transport (*rkp*Z1, *rkp*Z2, *rkp*R, *kps*F2, *kps*T, *kps*M) and predicted calcium-binding proteins (SMb20829, SMb20838), together with several conserved hypothetical proteins (Table 1). The largest region missing from the whole of the *S. meliloti* AK21 genome was found in pSymA (Table 1), which has also been partially lost from *S. meliloti* SM11 (pSymA cluster I, 17 kb). As for the losses from the chromosome, it was difficult to highlight a particular gene network, comprising metabolic genes, transporters and regulatory elements. The variation observed was probably induced by genetic exchanges mediated by mobile elements, such as insertion sequences or phages.

**Table 1:** Detailed description of *S. meliloti* 1021 gene clusters missing from the *S. meliloti* AK21 genome

Gene Clusters	Size (kb)	Gene ID^a^	Number of genes	Annotated functions
C1	19.6	SMc02187–SMc02151	14	Conserved hypothetical proteins; helicase DNA-binding protein
C2	18.1	SMc02326–SMc02303	18	Restriction-modification system (*hsdM/*S/R)
C3	10	SMc00267–SMc00276	10	Dehydrogenase; transketolase; transporter system; transcriptional regulator
C4	7.8	SMc00504–SMc00497	8	Transcriptional regulator; putative transmembrane proteins
C5	11.6	SMc01741–SMc01554	13	Conserved hypothetical proteins; acyl transferases; acyl carrier protein
C6	79.1	SMc03246–SMc03769	63	Temperate phage insertion; peptide/amino-acid metabolism and transport
A1	97.3	SMa0298–SMa0476	94	Transcriptional regulator; amino acid metabolism; deshydrogenase; ABC transporter systems; adenylate/guanylate cyclase (*cya*I2)
A2	92.8	SMa0947–SMa1141	107	ABC transporter systems; adenylate/guanylate cyclase (*cya*P); regulatory elements; cation transport
A3	66	SMa2113–SMa2245	59	ABC transporter systems; dehydrogenases; oxidoreductases; aminotransferases
A4	15.4	SMa2337–SMa2341	11	Rhizobactin biosynthesis and transport
B1	21.2	SMb20823–SMb20841	22	Capsular biosynthesis and export

^a^Gene identification according to the annotated genome of *S. meliloti* 1021.

### Gene expression profile of *S. meliloti* AK21

**Figure 2:**
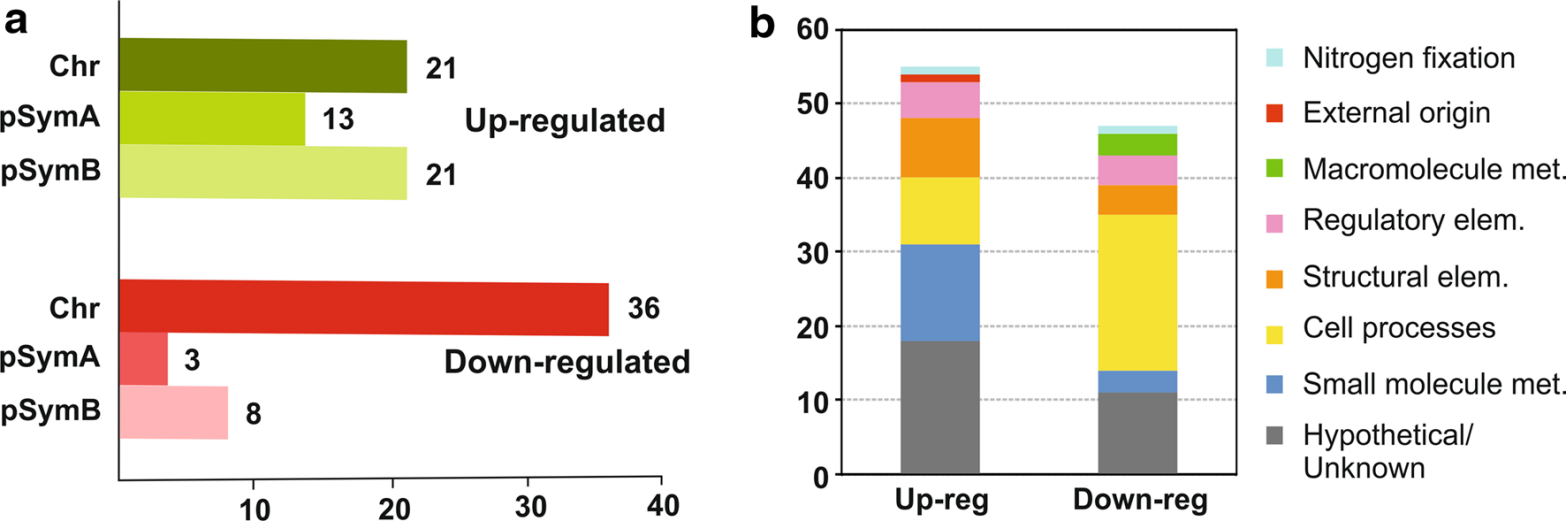
Distribution of genes differentially regulated in *S. meliloti* AK21 respect to the reference Rm1021. **a** Differences in color intensities are used to indicate the three replicons: chromosome (*darkest*), pSymA (*medium intensity*) and pSymB (*lightest*). **b** Functional distribution of the *S. meliloti AK21* differentially expressed genes according to the KEGG and *S. meliloti* databases functional categories. *Up-reg* up-regulated genes, *Down-reg* down-regulated genes.

*Sinorhizobium meliloti* AK21 was isolated from nodules of *Medicago sativa* L. subsp. *ambigua* growing in contaminated and saline soil (0.15 dS/m, about 0.8% NaCl). Therefore, we investigated whether differences in gene expression could contribute to the adaptation of *S. meliloti* AK21 to survive in these soils. We compared transcript abundances in *S. meliloti* AK21 grown into exponential growth phase (OD_600_ = 0.6) in TY rich medium with the expression profile of Rm1021 grown in rich medium to the same growth phase by cDNA hybridization on whole-genome Sm14kOligo microarrays. Hybridization signals corresponding to the intergenic regions were not considered in this analysis. We obtained 1,446 statistically significant expression signals for Rm1021/AK21 cross-hybridization, but only 102 genes were considered to be differentially expressed (|M| ≥ 1, see Additional file 2). In total, 47 of these genes were less strongly expressed in *S. meliloti* AK21 than in Rm1021, and 55 genes were more strongly expressed in *S. meliloti* AK21 (Figure 2a). The genes down-regulated in *S. meliloti* AK21 contained a particular high proportion of chromosomal genes (76.6% chromosome, 17% pSymB and 6.4% pSymA). By contrast, the up-regulated genes seemed to be much more evenly distributed between the three replicons (38.2% chromosome, 38.2% pSymB and 23.6% pSymA). From a functional perspective (Figure 2b; Additional file 2), we found that genes related to phosphate uptake were largely repressed in *S. meliloti* AK21 (*pst*S, *pho*C, *pho*D and *pho*E), as well as flagella-related genes and some other involved in chemotaxis. Moreover, we detected lower expression levels of the genes coding for the RNA polymerase sigma factor RpoE1 (SMc01418 to SMc01421). On the contrary, membrane components, including transporters and transmembrane proteins, were highly represented between the *S. meliloti* AK21 up-regulated genes. Interestingly, *fix* I1/P1/Q1/N1 (2.05/2.9/4.1/2.23-fold change, respectively) and *fix*Q2 (3.2-fold induction) exhibited increased expression in *S. meliloti* AK21 compared to Rm1021 (Additional file 2). Finally, a substantial number of hypothetical/unknown function proteins were found to be differentially regulated (29 of the 102 differentially expressed genes).

Taken together, our data point out that pSymA displayed only a few differences in gene expression despite the high level of plasticity inferred from its genetic structure. Conversely, high levels of variation were observed for the expression of genes on the chromosome.

### Suppression subtractive hybridization identifies different groups of genes differing with respect to the Rm1021 genome

**Figure 3:**
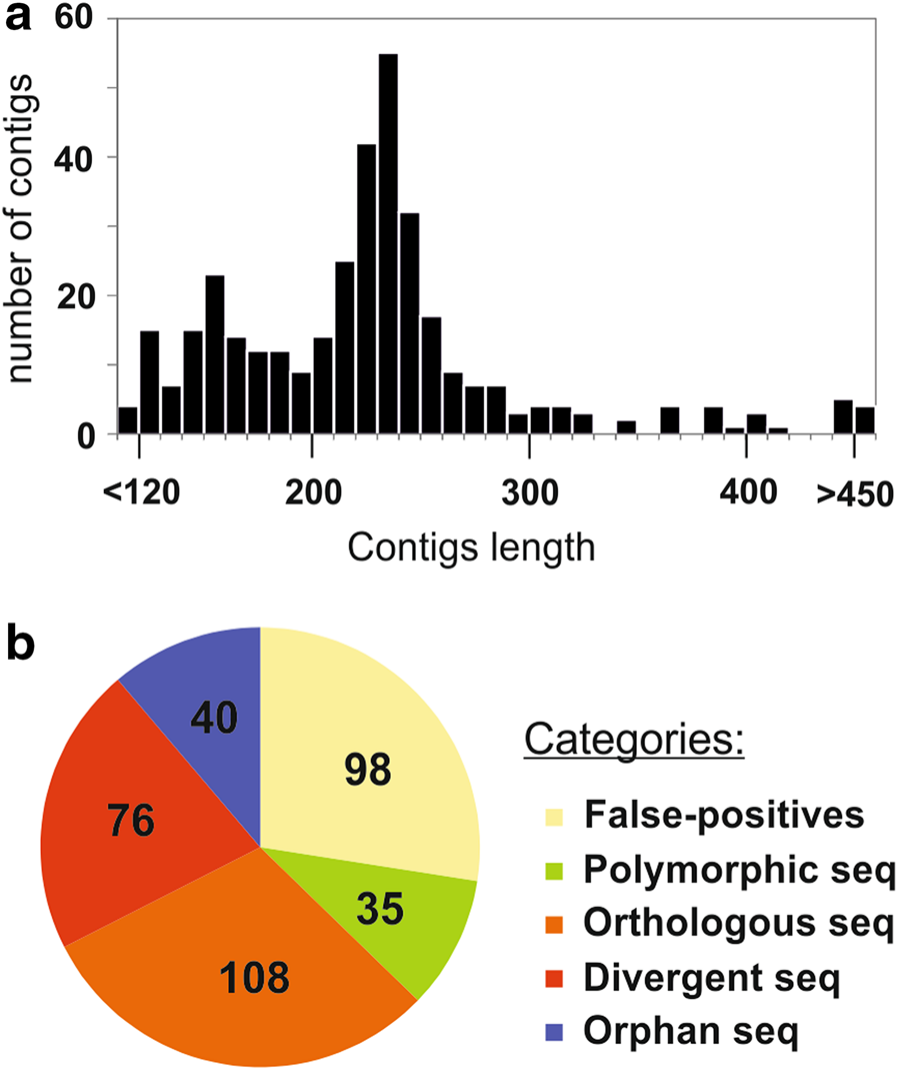
Characterization of suppression subtractive hybridization sequences. **a** Graphical representation of the number of assembled contigs (*Y* axis) against their length in base pairs. **b** Distribution of the different groups of sequences classified according to BLAST+ identification (NCBI, May, 2013). We distinguished five different categories: false-positives, which were 100% identical to sequences within the Rm1021 genome; polymorphic sequences, which had different nucleotide sequences but encoded the same protein sequence; orthologous sequences, which displayed >85% identity to *S. meliloti* 1021 proteins; divergent sequences, corresponding to genes present in the databases but displaying significant identity to sequences from bacteria other than Rm1021; and orphan sequences, not found in the databases.

Comparative genome hybridization is a powerful tool for identifying the genes common to two genomes, such as those of Rm1021 and *S. meliloti* AK21, but it could not provide information about the genes present exclusively in *S. meliloti* AK21. We therefore carried out suppression subtractive hybridization, by the selective enrichment of DNA sequences present in our tester strain, *S. meliloti* AK21, but not in the reference strain, Rm1021. The 454 FLX pyrosequencing of the differential fragments from *S. meliloti* AK21 resulted in 14,588 cleared reads, most of which (98.4%) were assembled into contigs with a mean length of 226 bp (Figure 3a).

In total, 357 contigs were generated, ranging from 119 to 640 bp in length. These sequences were then grouped into five categories (Figure 3b; Additional file 3): (a) false-positives displaying 100% identity to the Rm1021 genome sequence on Blastn analysis; (b) polymorphic sequences with slightly different nucleotide sequences but encoding proteins with sequences identical to those of the reference strain, as shown by Blastx analysis (100% identity); (c) orthologous sequences, encoding proteins displaying >85% identity to *S. meliloti* 1021 proteins on Blastx analysis; (d) divergent sequences, including those excluded from the previous categories, but for which homologous sequences were identified in the NCBI database by either Blastn or Blastx analysis; and finally, (e) orphan sequences displaying no identity to any of the known sequences in the databases. More than 25% of the sequences were false-positives, consistent with previous observations (Guo et al. 2005; Hermans et al. 2005; Zhang et al. 2005). The discriminative capacity of this technique made it possible to identify 40% of the total number of contigs as polymorphic or orthologous sequences. Most of the orthologous sequences displayed higher levels of identity to genes identified in *S. meliloti* strains other than Rm1021, including even the related but divergent species *S. medicae* WSM419. The number of orthologous sequences (50 contigs out of 108 analysed sequences) was higher for the pSymA megaplasmid than for the chromosome (33 contigs) or pSymB (25 contigs), consistent with the differences in variability between the three replicons described above (Additional file 3). Furthermore, this relative abundance of such sequences on pSymA may account for the large number of genes related to symbiosis (*noe*B) and nitrogen fixation (such as *arc*B, *nol*G and *nif*A) obtained in orthologous sequence identification. Verification of the flanking regions of some of the abovementioned orthologous sequences by the Southern blotting of restriction enzyme-digested total DNA revealed a similar genetic organization in the *S. meliloti* AK21 and Rm1021 genomes (data not shown). The orphan category comprised 40 of the 357 contigs, including a potentially significant number of undiscovered genes.

**Figure 4:**
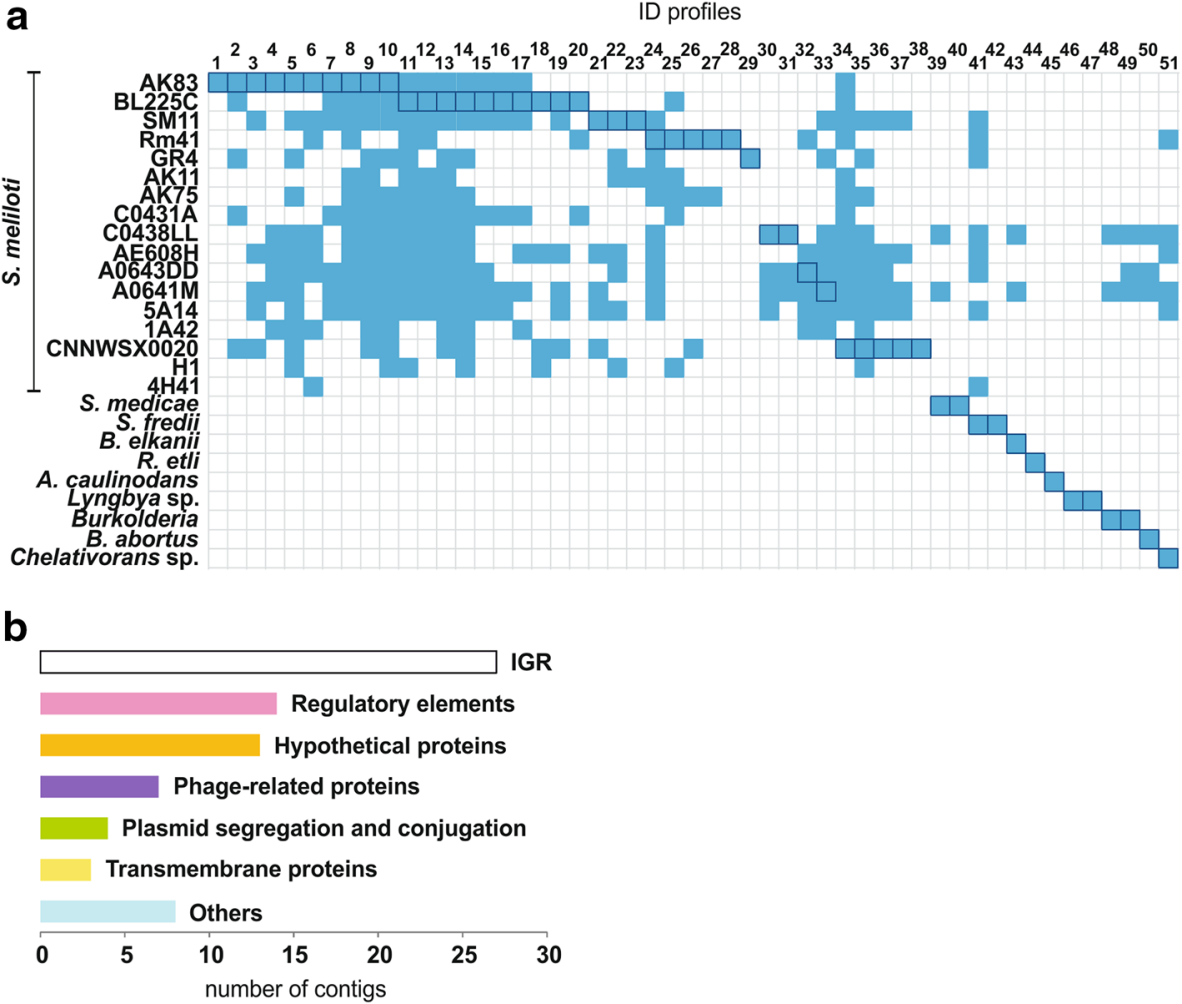
Analysis of the divergent sequences found by SSH. **a** The distribution of the divergent sequences among strains obtained in the BLAST+ analysis is shown. Identity (ID) profiles correspond to contigs which exhibit the same distribution among the different bacteria. *Shaded squares* indicate that the corresponding contig was totally or partially identical to a sequence present in the bacterium concerned. *Highlighted squares* identify the first entry in the BLAST results, corresponding to the sequence for which identity was the highest. The number of the contigs included in the different ID profiles is defined in additional file 3. **b** The most representative functions associated with the divergent sequences identified are plotted.

Finally, 76 of the 357 contigs were identified as divergent sequences, the distribution of which is shown in Figure 4a, after grouping in identity profiles according to its presence in the bacterial strains. *S. meliloti* was the most frequently identified species, particularly the wild strains AK83 and BL225C. A minor group of contigs displayed identity to sequences from other symbiotic rhizobacteria, such as *S. medicae*, *S. fredii*, *R. etli*, *Azorhizobium caulinodans* and *Bradyrhizobium elkanii* or, even pathogenic bacteria, such as *Brucella*. Only five contigs displayed identity with more distantly related bacteria: *Lyngbya* sp. (2), *Burkolderia* (2) and *Chelativorans* sp. (1). Functional analysis of this category revealed that the vast majority of the sequences corresponded to intergenic regions (IGR) and hypothetical proteins that could not be precisely classified (Figure 4b). The other large functional group of proteins matched diverse regulatory elements, including various families of transcriptional regulators (LysR-, AraC- and GntR-like regulators), adenylate/guanylate cyclases and a sequence which could encodes an autoinducer synthesis protein with the Sec-C regulatory motif. Several phage-encoded proteins and toxin-antitoxin systems were also identified, adding to the long list already reported for *S. meliloti* (Bodogai et al. 2006; Sharma et al. 2008). We also found sequences displaying identity to genes involved in the biosynthesis of rhizobitoxine (RtxA), a bacterial peptide that enhances host legume nodulation by inhibiting ethylene synthesis (Sugawara et al. 2006). Furthermore, proteins putatively involved in plasmid segregation (RepA and RepB, encoded by the accessory plasmid pSmeSM11a in *S. meliloti* SM11; Stiens et al. 2006) and conjugative transfer (VirB9-like protein and TraG) were partially sequenced. The group of divergent sequences was completed by genes encoding putative transmembrane proteins and metabolic enzymes, such as monophenol monooxygenase, which is involved in melanin synthesis. These results provide evidence of the presence of an accessory plasmid in *S. meliloti* AK21, potentially responsible for a battery of new activities relating to some extent to nodulation efficiency or survival in contaminated soils. More detailed analyses of these genes would provide us with insight into the processes in which these activities are involved and their link to the phenotypic advantages observed in *S. meliloti* AK21.

### Phenotypic microarrays identify interesting metabolic pathways in *S. meliloti* AK21

We compared the metabolic capabilities of *S. meliloti* AK21 with those of the reference strain Rm1021, with a phenotypic microarray (PM, Bochner et al. 2001). We measured the effects on bacterial respiration rates of adding various sources of carbon (PM1, PM2), nitrogen (PM3, PM6, PM7, PM8), phosphorus and sulfur (PM4) to the culture medium. We also investigated nutrient stimulation (PM5), osmotic and pH tolerance in terms of growth (PM9, PM10) and chemical sensitivity (PM11–20). In total, we studied 947 different growth conditions and determined resistance to different concentrations of 240 chemical reagents, including antibiotics, xenobiotics, fungicides and contaminants. We observed differences in growth rate between the strains in 28 of the 1,907 assayed conditions, corresponding to metabolic advantages of *S. meliloti* AK21 over Rm1021 (Table 2; Additional file 4). When 2-deoxy-d-ribose was added as a sole carbon source, *S. meliloti* AK21 grew better than Rm1021. Therefore with the exception of the use of 2-deoxy-d-ribose, the carbon use pathways of the two strains are similar. Strikingly, enhanced respiration rates were observed when most cyclic nucleoside monophosphates (cUMP, cCMP and cGMP) and some nucleoside monophosphates (CMP and UMP) were used as the phosphorous source. Sulfur phenotypes were also gained, with growth being sustained by l-methionine sulfoxide, methane sulfonic acid, *N*-acetyl-d,l-methionine, l-cysteic acid and cysteamine as the sole source of this non-metallic element. In general, *S. meliloti* AK21 seemed to make better use of diverse sources of P and S for growth than did other *S. meliloti* strains. However, it metabolized cysteine derivatives and methane sulfonic acid less efficiently (Biondi et al. 2009). It also displayed higher respiration rates in the presence of some oligopeptides: leu-leu, gly-asn, lys-asp, val-phe and val-tyr-val. *S. meliloti* AK21 grew at pH 10 and in the presence of 5% potassium chloride, unlike other *S. meliloti* isolates (Biondi et al. 2009). Several drugs were identified as growth-enhancing compounds, affecting nucleic acid metabolism (pipemidic acid, lomefloxacin and rifampicin), amino acid metabolism (benserazide and tylosin), phosphate metabolism (X-PO_4_ and sodium orthovanadate), and pesticide tolerance (tolylfluanid, domiphen bromide and lawsone). The tolerance of *S. meliloti* AK21, in terms of growth, may therefore reflect the adaptation of this strain to an adverse environment, containing high levels of fertilizer, salt and other chemical contaminants.

**Table 2:** Metabolic capabilities acquired by *S. meliloti* AK21, as identified on phenotypic microarrays

Tested compound	Average height^a^	Metabolic process
2-Deoxy-d-ribose	41	C-Source, carbohydrate
Leu-Leu	61	N-Source, peptide
Val-Tyr-Val	51	N-Source, peptide
Gly-Asn	43	N-Source, peptide
Lys-Asp	43	N-Source, peptide
Val-Phe	40	N-Source, peptide
Uridine 2′,3′-cyclic monophosphate	53	P-Source, organic
Cytidine 2′,3′-cyclic monophosphate	46	P-Source, organic
Guanosine 2′,3′-cyclic monophosphate	43	P-Source, organic
Cytidine 3′-monophosphate	41	P-Source, organic
Uridine 3′-monophosphate	40	P-Source, organic
l-methionine sulfoxide	52	S-Source, organic
Methane sulfonic acid	52	S-Source, organic
*N*-Acetyl-d,l-Methionine	45	S-Source, organic
l-Cysteic acid	44	S-Source, organic
Cysteamine	42	S-Source, organic
5% potassium chloride	61	Osmotic sensitivity, KCl
pH 10	59	pH, growth at 10
X-PO4	51	Aryl phosphatase
Benserazide	96	aa metabolism, aromatic amino acid decarboxylase inhibitor
Tylosin	55	Protein synthesis, 50S ribosomal subunit, macrolide
Rifampicin	126	RNA polymerase
Pipemidic acid	106	DNA topoisomerase
Lomefloxacin	72	DNA topoisomerase
Sodium orthovanadate	78	Toxic anion, PO_4_ analog
Lawsone	93	Oxidizing agent
Domiphen bromide	76	Membrane, detergent, cationic, fungicide
Tolylfluanid	193	Fungicide, phenylsulfamide

^a^Average height is the area under the curve divided by number of measurements.

## Discussion

*Sinorhizobium meliloti* AK21 was isolated from a highly polluted and salinized, dry soil in the northern Aral Sea Region. We compared the genomes of *S. meliloti* AK21 and the sequenced strain *S. meliloti* 1021, and analysed differential gene expression and carried out phenotypic characterization. We found that 364 out of 365 differing genes of *S. meliloti* 1021 were missing from *S. meliloti* AK21, and that these genes were mainly clustered in 11 regions of variable length, generally flanked by mobile elements and also by conserved sequences with a complex secondary structure. Genetic variations were most frequently found to genes located on the megaplasmid pSymA of Rm1021, followed by the chromosome and pSymB. We also detected a set of genes in *S. meliloti* AK21 that were polymorphic or orthologous to genes in other rhizobia, and divergent sequences that were present in more phylogenetically distant bacteria. The gene expression profile revealed differences in regulation between free-living *S. meliloti* AK21 and Rm1021, with a greater number of genes up-regulated than down-regulated relative to Rm1021. Finally, *S. meliloti* AK21 grew in various limiting conditions, although the genes involved in these new capabilities have yet to be determined.

Rhizobial genomes are usually partitioned in several replicons, the number of which depends on the species; though additional, small replicons could be present in different isolates. The chromosome and the pSymB chromid display almost perfect correlation for the presence of genes, with only a few inserted, deleted or rearranged regions (Galardini et al. 2011). However, the symbiotic megaplasmid pSymA, and the accessory plasmids described in rhizobia, have a higher frequency of genomic rearrangements, making them dynamic structures prone to genome evolution and adaptation (Mavingui et al. 2002; Giuntini et al. 2005; Stiens et al. 2006; Galardini et al. 2011, 2013b; Bailly et al. 2011). Our comparative genomic hybridization and suppressive subtraction hybridization data support these previous findings, because the longest regions lost and the highest proportion of polymorphic and orthologous genes mapped to pSymA of Rm1021. The proportion of variable genes in the whole genome of *S. meliloti* AK21 was about 5.8%, similar to the results reported for previous studies of other *S. meliloti* strains (Giuntini et al. 2005; Stiens et al. 2008). However, most of this variability (3.6%) was associated with pSymA, followed by the chromosome (1.7%) and pSymB (0.5%). An in silico analysis comparing our CGH results and the whole-genome data for a collection of recently sequenced *S. meliloti* strains revealed that most of the gene clusters missing from the genome had also been lost from these other strains, suggesting that these regions were potentially acquired by Rm1021, rather than lost from *S. meliloti* AK21 (see Additional file 5). We found that almost 20% of the pSymA genes were missing from *S. meliloti* AK21 or differed substantially from those found in Rm1021. In addition, insertion sequences and transposons have frequently been found on pSymA-type plasmids (Barloy-Hubler et al. 2000; Barnett et al. 2001; Galibert et al. 2001; Schneiker-Bekel et al. 2011). Thus, these plasmids would be expected to display higher rates of rearrangement, due to the activity of these mobile elements. Indeed, the rhizobial symbiotic clusters, generally linked to pSymA, is generally thought to have been acquired by horizontal transfer between different species, and even genera, because *nif*-*nod* regions are surrounded by transposases and repeated sequences (Sullivan and Ronson 1998; Mavingui et al. 2002; Young et al. 2006; Crossman et al. 2008; Galardini et al. 2011; Bailly et al. 2011; Epstein et al. 2012). Most transposase-encoding genes are found in regions carrying traces of genomic rearrangements (Galardini et al. 2011). The core genome is generally considered to define bacterial phylogeny, whereas the accessory genes shed light on biological aspects of the symbiosis (Young et al. 2006; Galardini et al. 2011, 2013b; Bailly et al. 2011; Epstein et al. 2012; Tian et al. 2012; Sugawara et al. 2013). Our results are consistent with this hypothesis, because the divergent sequences obtained by SSH mostly matched with *S. meliloti*, mainly with *S. meliloti* AK83, which was isolated from the same ecological niche as *S. meliloti* AK21.

The major differences in gene expression between Rm1021 and *S. meliloti* AK21 concerned membrane components. Flagellum genes were much less strongly expressed in *S. meliloti* AK21 than in Rm1021. In addition, previous studies on *S. meliloti* 1021 reported the down-regulation of a number of genes involved in chemotaxis and cell motility after an osmotic upshift or during osmoadaptation (Rüberg et al. 2003; Domínguez-Ferreras et al. 2006). Flagella are usually linked to cell motility, unless flagellum-independent bacterial movement has also been described (Nogales et al. 2012), and the correct regulation of flagellum movement is essential for legume nodulation (Soto et al. 2002). In *S. meliloti*, surface motility is regulated by ExpR, in conjunction with the Sin-AHLs quorum-sensing system, in different manners, depending on cell density (Hoang et al. 2008; Nogales et al. 2012). At high population densities, both regulatory systems inhibit the flagellar regulon through the *vis*NR transcriptional regulator. *S. meliloti* AK21 is an ExpR-proficient bacterium producing large amounts of capsular polysaccharide, whereas Rm1021 is a nonmucoid strain in which an insertion sequence interrupts the coding sequence of *exp*R (Pellock et al. 2002). We obtained indirect evidence for the production of flagella in *S. meliloti* AK21, which displayed swimming motility, which is strictly dependent on flagella (data not shown). Consistent with this finding, *S. meliloti* AK21 does not probably swarm in semisolid agar plates, instead moving over the surface by sliding (data not shown). Rhizobactin 1021 is a siderophore that is thought to be required for surface mobility in Rm1021 (Nogales et al. 2010, 2012). According to our CGH data, *S. meliloti* AK21 seems to lack the rhizobactin biosynthesis operon, suggesting that this sliding motility is probably rhizobactin-independent. However, more detailed studies of flagellum production and surface motility are required to obtain insight into these two processes in *S. meliloti* AK21. Furthermore, the determination of *S. meliloti* genome expression profiles led to the identification of a set of ExpR-controlled genes involved in other important cellular processes, such as nitrogen fixation, metabolism and metal transport (Hoang et al. 2004). As we compared an ExpR-deficient strain (Rm1021) and *S. meliloti* AK21, a strain that can potentially produce this regulatory protein, it is not surprising that a detailed analysis of our data revealed several genes previously reported to belong to the ExpR/Sin operon. Similarly, we also identified several genes related to the PhoB regulatory network, which was found to be downregulated in the *S. meliloti* AK21 transcriptomic dataset (19 of 102 differentially expressed genes). PhoRB is a two-component regulatory system involved in diverse cellular processes, such as exopolysaccharide biosynthesis, de novo NAD biosynthesis, biofilm formation and secondary metabolite production, but its most frequently studied functions in *S. meliloti* relate mostly to phosphate metabolism and starvation (Krol and Becker 2004; Yuan et al. 2006). However, these authors reported differential regulation of this two-component system in Rm1021 and Rm2011, and concluded that Rm1021 displayed moderate levels of PhoB activation, even in the presence of sufficient phosphate, due to a deletion in the *pst*C gene from Rm1021. Therefore it is possible that much of the differential expression observed between *S. meliloti* AK21 and Rm1021 is due to PhoB-dependent regulation. In summary, since there seems to be an ExpR and PhoB differential regulation between Rm1021 and *S. meliloti* AK21, we have to be cautious to interpret the microarrays expression data.

Finally, FixQ1 and FixQ2 were found to be induced in *S. meliloti* AK21 grown in standard conditions, that is to say rich medium and aerobiosis. The *fix*NOQP genes encode the membrane complex cytochrome *cbb*3 oxidase, a final electron acceptor in the respiratory chain, which has a high affinity for O_2_ under the low oxygen concentrations found in most rhizobia (i.e. in symbiosis with plants) (Becker et al. 2004; Torres et al. 2013). FixQ seems to be a structural element that stabilizes the interaction of CcoP with the CcoNO core complex (Peters et al. 2008). The FixLJ/FixK regulatory proteins control the expression of *S. meliloti* genes required for nitrogen fixation and for microaerobic respiration within nodules, including the *fix*NOQP1 and two operons strongly induced under low-oxygen conditions in free-living bacteria and in bacteroids, respectively (Bobik et al. 2006). The *fix*N1, *fix*N2, *fix*I and *fix*P genes were also identified as induced, but to a much lesser extent than *fix*Q. We observed a slight repression of *fix*J, *fix*K1 and *fix*K2, whereas other genes from the FixLJ/FixK regulon were found to be differentially regulated (induced or repressed, depending on the gene). These data suggest that *fix*NOQP could be constitutively expressed in *S. meliloti* AK21 and could be related to the stress response, whereas in Rm1021 is under the tight control of FixLJ/FixK. The induction of cytochrome *cbb*3 may increase the ATP content of the bacterium in preparation for the establishment of symbiosis, considering the key role of *fix*NOQP1 in early stages of association with plants (Torres et al. 2013). This protein complex may therefore play a role in the adaptation of *S. meliloti* for nodulation in saline conditions. These results suggest an alternative pathway for the activation of the *fix* genes in response to the presence of salt and/or other chemical compounds in the environment.

In terms of phenotypic adaptations, we observed that *S. meliloti* AK21 is able to efficiently support growing in a wide variety of S, N and P sources as well as in contaminated environments. Similar studies carried out with bacteria isolated from the same region, *S. meliloti* AK83 or AK58, revealed that these natural isolates, mainly AK58, are able to proliferate in different C and N sources and in a plethora of antibiotics and other chemicals (Biondi et al. 2009; Galardini et al. 2014). However, these phenotypes are not shared with *S. meliloti* AK21. It is likely that each organism could acquire particular abilities for surviving in hostile environments. Conversely, Rm1021 has been grown in laboratory conditions for a long period and does not require these metabolic adaptations.

ARDRA analysis and 16S rRNA gene sequencing classified AK21 as *S. meliloti*. Eckhardt lysis analysis of plasmid content revealed the presence of three major replicons, probably corresponding to the chromosome, pSymA and pSymB, and a cryptic plasmid of about 120 kb in size (Ibragimova et al. 2006; M. Bazzicalupo personal communication). Our SSH data are consistent with this experimental evidence, because we found proteins involved in plasmid replication/partitioning from the *rep*ABC family to be exclusive to *S. meliloti* AK21. Moreover, we identified several genes usually carried by accessory plasmids, including a putative mobilization module TraG/Vir (pSmeSM11a) and a monophenol monooxygenase gene (pRmeGR4b). These accessory plasmids carry genes that could confer an adaptive advantage on the host bacterium and they are widespread and interchangeable between natural rhizobial populations (more than 50% of rhizobia harbor at least one cryptic plasmid; Mercado-Blanco and Toro 1996; Stiens et al. 2006; Galardini et al. 2013b). Thus, the rhizobitoxine biosynthesis operon identified in *S. meliloti* AK21 may be present on the accessory plasmid. This phytotoxin was first described in *Bradyrhizobium* species as being associated with leaf chlorosis in susceptible host plants (Ruan and Peters 1992; Ruan et al. 1993). It is also produced by other pathogenic microorganisms, such as *Pseudomonas* and *Agrobacterium*, and has recently been found in a group of *S. medicae* isolates (Bailly et al. 2011). Rhizobitoxine interferes with ethylene synthesis in plant tissues, by inhibiting the ACC synthase (Yuhashi et al. 2000; Sugawara et al. 2006). Likewise, the *acd*S gene has been shown to be present on pSmeSM11a, one of the accessory plasmids of *S. meliloti* SM11 (Stiens et al. 2006). It encodes an ACC deaminase that metabolizes the precursor of the phytohormone ethylene, thereby enhancing nodulation (Ma et al. 2004). Similar strategies for the improvement of nodulation, through the inhibition of ethylene biosynthesis, could thus be mediated by accessory genes, conferring fitness advantages for the symbiotic interaction. The presence of these genes on transmissible plasmids would facilitate spread within the population of bacteria in the soil.

## Conclusions

Rhizobiaceae is a family of bacteria of enormous agricultural importance, due to their ability to fix atmospheric nitrogen in symbiosis with leguminous plants (Jones et al. 2007). They occur naturally in most agricultural soils, but fluctuations in environmental conditions, such as temperature, osmolarity, pH or nutrient availability, severely affect their symbiotic capacity (Zahran 1999; Rüberg et al. 2003). For this reason, studies of the mechanisms by which indigenous populations of rhizobia adapt to unbalanced environments are required. *S. meliloti* AK21 was isolated from highly degraded and saline soils in the Aral Sea Region, in which it efficiently nodulates *M. sativa*. In this study, we demonstrated the existence of genetic, transcriptomic and phenotypic differences between this wild strain and the laboratory reference strain, *S. meliloti* 1021. The chromosome of *S. meliloti* AK21 had lost six regions, but only minor differences in gene expression were observed. Similarly, the symbiotic plasmid pSymA lacked four regions, and three of these deletions were extensive. However, the largest differences in gene expression were found for this replicon. Conversely, the chromid pSymB lacked only one short region. A significant number of these deleted gene clusters were flanked by transposable and repeated elements that could potentially be involved in homologous recombination. We also identified several sequences that were absent from *S. meliloti* 1021 but present in other rhizobia, and sequences present exclusively in *S. meliloti* AK21. These groups of divergent and orphan contigs may account for the phenotypic advantages of the wild isolate over the reference strain. Moreover, we detected several proteins involved in plasmid conjugation and segregation, and several genes also reported to be present on accessory plasmids in other *S. meliloti* strains. Then, we obtained genetic evidence for the presence of an accessory plasmid in *S. meliloti* AK21. Finally, we discovered a large set of genes differentially expressed between *S. meliloti* AK21 and Rm1021. Membrane components predominated over all other cellular functions, but we detected two proteins related to nitrogen fixation, FixQ1 and FixQ2. These proteins form part of the *cbb*3 electron transport system and may be involved in enhancing the nodulation behaviour of rhizobia in saline conditions.

## Methods

### Bacterial strains and culture conditions

*Sinorhizobium meliloti* 1021 (Rm1021) forms nitrogen-fixing nodules in *Medicago* hosts. This streptomycin-resistant derivative of *S. meliloti* SU47 has been grown under laboratory conditions for 38 years (Meade and Signer 1977). Rm1021 was the first *S. meliloti* strain to be sequenced and is therefore considered to be the reference strain for this species. *S. meliloti* AK21 was isolated from nodules of *Medicago sativa* L. subsp. *ambigua* (formerly named *M. trautvetteri*) growing in Kazakhstan, in the northern Aral Sea Region (State Research Center of the Russian Federation Collection in St. Petersburg; Ibragimova et al. 2006). Rhizobia were cultured at 28°C in rich TY broth (5 g/l tryptone, 3 g/l yeast extract, 0.9 g/l CaCl_2_), with constant shaking at 180 rpm.

### Isolation of *S. meliloti* genomic DNA

DNA was extracted with the RealPure genomic DNA extraction Kit (Durviz, S.L.U., Valencia, Spain), according to a modified version of the procedure recommended by the manufacturer. Briefly, pelleted cells were washed with 0.1% l-laurylsarcosine in TE buffer, pH 8. The cells were lysed by incubation for 5 min at 80°C and then subjected to RNAse treatment at 37°C for 60 min. RNA and proteins were removed, and the DNA was precipitated with 2-propanol. Purified DNA was quantified spectrophotometrically (Nanodrop, PeqLab, Erlangen, Germany). Highly pure genomic DNA (*A*260/*A*280 ratio of 1.8–2.0) was obtained at a final concentration of about 1.2–1.4 μg/μl.

### Total RNA extraction from *S. meliloti*

High-quality total RNA was isolated with the RNeasy Mini Kit (Qiagen, Barcelona, Spain), according to a modified version of the manufacturer’s protocol. We harvested six OD_600_ units of bacterial cultures in the exponential growth phase by rapid centrifugation and froze them in liquid nitrogen. The samples were then thawed on ice, and the cells were resuspended in 100 μl of freshly prepared lysozyme solution (0.4 mg/ml) and the samples were incubated at room temperature for 5 min. The bacteria were then disrupted by adding 350 μl of RLT buffer and shaking vigorously, and the disrupted cells were then centrifuged for 2 min. The cleared lysates were mixed with 250 μl of 100% ethanol, transferred to the supplied spin columns and centrifuged. The columns were then washed according to the manufacturer’s instructions and the RNA was eluted in 120 μl of water. All trace DNA contamination was removed by DNA digestion with 30 units of RNAse-free DNAse I (Qiagen, Barcelona, Spain) at 28°C for 90 min. We then added 490 μl of RLT buffer and 350 μl of 100% ethanol to the samples and the mixture was layered onto a new column. The RNA was eluted in 50 μl MilliQ water, and further purified and concentrated on Microcon 30 columns (Millipore Iberica S.A.U., Madrid, Spain). RNA concentration was measured with a Nanodrop spectrophotometer and RNA quality was checked by gel electrophoresis.

### Comparative genome hybridization and data analysis

For genomic comparisons, we used whole-genome Sm14kOligo microarrays supplied by the Center for Biotechnology, University of Bielefeld, Germany. These microarrays carry 50-mer to 70-mer oligonucleotide probes directed against coding regions and intergenic regions (see http://www.cebitec.uni-bielefeld.de/transcriptomics/transcriptomics-facility/sm14koli.html for further details). Competitive hybridization was carried out twice for each restriction enzyme and strain, with dye swapping between the test and reference strains. Total DNA was labeled with the Bioprime Total Genomics Labeling System (Invitrogen, Madrid, Spain), as previously described by Giuntini et al. (2005). In brief, we labeled 10 µg of *Rsa*I- or *Taq*AI-digested genomic DNA with dCTP coupled to Alexa Fluor 3 or Alexa Fluor 5 in a 50 μl reaction mixture containing random primers and the Klenow fragment of DNA polymerase I, by incubation at 37°C for 2 h. The unincorporated fluorescent nucleotides were removed and the samples were combined as appropriate and mixed with 30 μl Cot-1 DNA (1 mg/ml), 17 μl yeast t-RNA (6 mg/ml) and 450 μl TE buffer. Microcon 30 filter columns (Millipore Iberica S.A.U., Madrid, Spain) were used to concentrate the probes down to a final volume of about 35 μl. Each combined sample was mixed with 8.5 μl of 20× SSC and 0.74 μl of 10  SDS and denatured by heating at 100°C for 1.5 min. The sample was then incubated at 37°C for 30 min and the hybridization probe was added to the pretreated microarray under a coverslip. Hybridization was performed at 65°C for 16 h, within a chamber in a water bath. The coverslip was removed by vigorous shaking in 2× SSC/0.2% SDS at 65°C for 30 s and slides were washed successively with: 2× SSC/0.2% SDS at 65°C for 5 min, 0.2× SSC/0.1% SDS at 65°C for 2 min and then twice with 0.2× SSC at room temperature, for 2 min each. The slides were immediately dried by centrifugation and the data were acquired on a GenePix 4000A scanner (Axon Instruments). Mean signal intensities for each spot were quantified with GenePix Pro 5.1 (Axon Instruments, Union City, CA, USA).

Raw data were normalized with MADE-4-2C (Fernandez-Pozo 2012), applying the LOESS scaled algorithm. Further statistical analysis of the normalized data using the same software allowed determining the differential presence of genes. A gene was considered to display a significant difference in hybridization if the intensity of the spot was outside the values included in the 95% confidence interval (*t* test, *p* value <0.05, corrected for multiple testing) and the absolute value for the log_2_-ratio of intensities was higher than 2. We obtained signal for 13,618 spots of the total 29,952 (total number of features). A positive log_2_-fold-change means that hybridization intensity was greater for *S. meliloti* AK21 than for Rm1021, indicating gene duplication. A negative log_2_-ratio of intensities indicated higher signal intensity for Rm1021, consistent with the corresponding gene being highly divergent in or missing from the *S. meliloti* AK21 genome.

### Microarray hybridization and statistical analysis of the data

We analysed the expression of the genes common to Rm1021 and *S. meliloti* AK21 by competitive cDNA hybridization on Sm14kOligo microarrays. The procedure was basically as previously described (Rüberg et al. 2003). In brief, 15 μg of total RNA was reverse-transcribed with 15 μg of random hexamer oligonucleotides, 400 U SuperScript II reverse transcriptase (Invitrogen, Madrid, Spain) and a 100 mM dNTP + aa-dUTP mixture (dNTPs: Roche Diagnostics, Barcelona, Spain; aa-dUTP: Sigma-Aldrich Quimica SL, Madrid, Spain) at 42°C for 2 h. After alkaline hydrolysis of the RNA, amino-allyl modified first strand cDNAs was cleaned using CyScribe GFX Purification Kit (GE Healthcare, Seville, Spain) and coupled with Cy3- and/or Cy5-NHS ester dyes (GE Healthcare, Seville, Spain) by incubation at room temperature for 2–3 h. The cDNA generated from Rm1021 RNA was labeled with Cy5 fluorescent dye, whereas the test cDNA from *S. meliloti* AK21 was coupled with the Cy3 fluorophore. Samples with different labels were mixed and the uncoupled dyes were removed with the CyScribe GFX Purification Kit (GE Healthcare, Seville, Spain). The dried probe was diluted in 45 μl DIG Easy Hyb solution (Roche Diagnostics, Barcelona, Spain) supplemented with 5 μg salmon sperm DNA. The denatured probe solution was poured onto the microarrays after the prehybridization step. Then, the microarrays were covered with cover slips and incubated in a hybridization chamber in a water bath at 42°C for at least 16 h. Microarrays were then washed at room temperature, as described above.

Data acquisition and processing are described in the section dealing with CGH. We analyse three biological and two technical replicates, swapping the dyes between the test and reference strains. Differentially expressed genes were identified on the basis of the following criteria: *p* value <0.05 (*t* statistics) and log2-ratio of intensities ≥1 or ≤−1. A positive value corresponds to genes more strongly expressed in *S. meliloti* AK21 than in Rm1021. Conversely, a negative ratio indicates that the corresponding genes are repressed or less expressed in *S. meliloti* AK21.

### Suppression subtractive hybridization and data processing

The PCR-Select Bacterial Genome Subtraction kit (Clontech, Madrid, Spain) was adapted for the detection of genes present in *S. meliloti* AK21, as the tester strain, but not in the driver strain *S. meliloti* 1021. We digested 2 µg each of the driver and tester genomic DNAs separately with *Rsa*I, to obtain short, blunt-ended fragments. The enzyme was removed and the digested tester DNA sample was split into two batches, each of which was ligated with a different, partially complementary adaptor supplied by the manufacturer, in the kit. These adaptors could bind to only one of the ends of the DNA fragments. Two successive hybridizations were then carried out to enrich the sample in tester-specific molecules absent from the reference genome. The adaptor-bound tester DNA and the *Rsa*I-digested driver DNA were denatured by heating at 98°C for 2 min, mixed in a 1:50 (tester/driver) ratio and renatured by heating at 64°C for 90 min. In a second hybridization step, the two previously annealed mixtures were combined and mixed with 300 ng of new freshly denatured driver genomic DNA. The reaction mixture was incubated at 64°C for 16 h. Two successive nested PCRs increased the enrichment of the sample in molecules flanked by a different adaptor. The second PCR products were sent for DNA pyrosequencing by 454 FLX method (Roche) at LifeSequencing SL (Valencia, Spain).

SeqTrim software was used to clean sequences and to remove adaptor sequences and PCR artifacts (Falgueras et al. 2010). The resulting sequences were assembled into contigs by CAP3 (Huang and Madan 1999). Two sequences were considered to constitute a single contig when they overlapped by at least 20 nt and overall identity exceeded 95%. Finally, BLAST + 2.2.25 (Blastn and Blastx) was used for sequence comparison and identification (Camacho et al. 2009). Blast2GO was used for the annotation of highly divergent sequences (Conesa et al. 2005) and orthologs were identified by the BBH method (Altenhoff and Dessimoz 2009). These sequences were uploaded as a library into the GSS database [LIBGSS: 038882].

### Phenotypic microarray analysis

*Sinorhizobium meliloti* AK21 and Rm1021 were assayed on phenotypic microplates (BiOLOG, Hayward, CA, USA) testing hundreds of different conditions. Runs were performed for the first twenty 96-well microplates available. Bacterial growth was measured in the presence of different C, P, N and S sources, fungicides and xenobiotic compounds, together with antibiotic resistance. Survival was also analysed in the presence of osmolytes and at different pH values (http://www.biolog.com/products-static/phenotype_microbial_cells_use.php). All the procedures were carried out by the manufacturer (BiOLOG, Hayward, CA, USA). *S. meliloti* cells were used to inoculate the wells of the array, which was then incubated at 30°C for 72 h in an OmniLog^®^ reader. The metabolic activities of each strain were evaluated in duplicates. Cell respiration rates were monitored colorimetrically over time and are presented as time-course graphs (Bochner et al. 2001). Pairwise comparisons were performed such that Rm1021 is shown in red and *S. meliloti* AK21 in green, with the overlapping areas shown in yellow. The OmniLog^®^ V. 1.5 Comparison Module provided a report consisting of a reproducibility test, the time-course plots and a list of compounds differentiating the metabolism of the test strain, *S. meliloti* AK21, from that of the reference strain, Rm1021. The reproducibility report indicated the number of wells for which the difference in the average height (area under the curve divided by the number of measurements) between duplicate runs was less than the threshold value (usually 12 wells).

## Additional files

**Additional file 1**: List of variable genes derived from CGH studies. The clusters of missing genes are shadowed and identified in the first column (with *p*-value < 0.01 and │logFC│ ≥ 2). The gene names and the corresponding log_2_-fold-change are indicated in the second and third columns. NCBI and Blast2GO descriptions are shown, together with statistical parameters.
**Additional file 2:** List of differentially expressed genes obtained by cDNA microarray hybridization. This list includes the raw data for the genes displaying significant differential expression (with *p*-value < 0.05) in Rm1021 versus *S. meliloti* AK21, both grown in TY medium (1021 *vs.* AK21). PhoB- and ExpR-regulated genes are indicated, in accordance with the findings of previous studies (Hoang et al. 2004; Krol and Becker 2004; Yuan et al. 2006).
**Additional file 3:** List of genes identified by SSH. Ordered register of the *S. meliloti* AK21 contigs obtained by the pyrosequencing of supressed samples. A complete description of the contigs and identities is provided, together with the category classification assigned. Gene identification, if possible, and the top-scoring organism are indicated in columns D and E, respectively. The specificity of the sequence for *S. meliloti* AK21 is indicated by the list of other organisms displaying identity to the query contig sequence presented in column F. Those found in Rm1021 were not analyzed (–NA–). Columns H to K include the identity index corresponding to: (**1**) the Blastn Rm1021 database; (**2**) the Blastx Rm1021 database; (**3**) the Blastn NCBI database (nt); and (**4**) the Blastx NCBI database (nr). Additional Blast2GO descriptions are shown for some of the sequences. A list of the contig profiles identified for Figure 4 is provided. Data updated in May, 2013.
**Additional file 4:** Phenotypic microarray-derived kinetic studies comparing *S. meliloti* AK21 with Rm1021. Two-run consensus curves correspond to the pairwise comparison between the test strain *S. meliloti* AK21 (in green) and the reference strain Rm1021 (in red). Boxed wells exceed the average height threshold in both of the independent experiments. Positive differences correspond to a gain of phenotype or resistance in the test strain with respect to the reference strain. The PM plate used are those with number PM-1 till PM-20 of the Biolog company (http://www.biolog.com/products-static/phenotype_microbial_cells_use.php).
**Additional file 5:** Comparative genomic analysis, comparing *S. meliloti* AK21 CGH data with data for other sequenced *S. meliloti* strains. Circular representation of the three main replicons of *S. meliloti*: chromosome, chromid pSymB and megaplasmid pSymA. The outer circle shows the genomic position, in Mb, according to the Rm1021 genome annotation. The next four concentric circles represent the missing genes in the analyzed *S. meliloti* genomes. The number of genomes that have lost the indicated genes is shown from the outer part of the diagram to the inner part, with five genomes in the first circle and 20 genomes in the last circle. Finally, the innermost group of circles indicate the GC skew determined for Rm1021. Highlighted regions in the outer circle correspond to the missing regions described in the main text and detailed in Table 1. The lower G + C content of Rm1021 corresponds to the regions missing from most of the *S. meliloti* genomes. The in silico comparison was carried out with BLAST + software and by BBH-based analysis (Camacho et al. 2009; Altenhoff and Dessimoz 2009). We created local protein databases for each genome, updated in May 2013, and established pairwise comparisons with Rm1021: Rm1021 [NCBI:PRJNA57603, PRJNA19]; SM11 [NCBI:PRJNA159685, PRJNA41117]; AK83 [NCBI:PRJNA52607, PRJNA41993]; 2011 [NCBI:PRJNA193772, PRJNA187276]; BL225C [NCBI:PRJNA52605, PRJNA42477]; GR4 [NCBI:PRJNA184823, PRJNA175860]; Rm41 [NCBI:PRJEA176372, PRJEB436]; 1A42 [NCBI:PRJNA199493, PRJNA167584]; 4H41 [NCBI:PRJNA199075, PRJNA169747]; 5A14 [NCBI:PRJNA199492, PRJNA167593]; A0641 M [NCBI:PRJNA199490, PRJNA167594]; A0643DD [NCBI:PRJNA199488, PRJNA167595]; AE608H [NCBI:PRJNA199486, PRJNA167596]; AK11 [NCBI:PRJNA199484, PRJNA167597]; AK75 [NCBI:PRJNA199482, PRJNA167598]; C0431A [NCBI:PRJNA199480, PRJNA167599]; C0438LL [NCBI:PRJNA199478, PRJNA167600]; CCNWSX0020 [NCBI:PRJNA180010, PRJNA75085]; and, H1 [NCBI:PRJNA199476, PRJNA167601]. A defined protein was considered to be lost if it met the following criteria: Blastp e-value ≤ 10^−6^; Blastp  % identity ≥ 90; Blastp matched length ≥ 85 % of the annotated gene length.
