# Supplementary figures and images for: Genomic characterization of Sinorhizobium meliloti AK21, a wild isolate from the Aral Sea Region
Source: Springerplus. 2015 Jun 16;4:259. doi: 10.1186/s40064-015-1062-z (PMC4468178; doi:10.1186/s40064-015-1062-z)

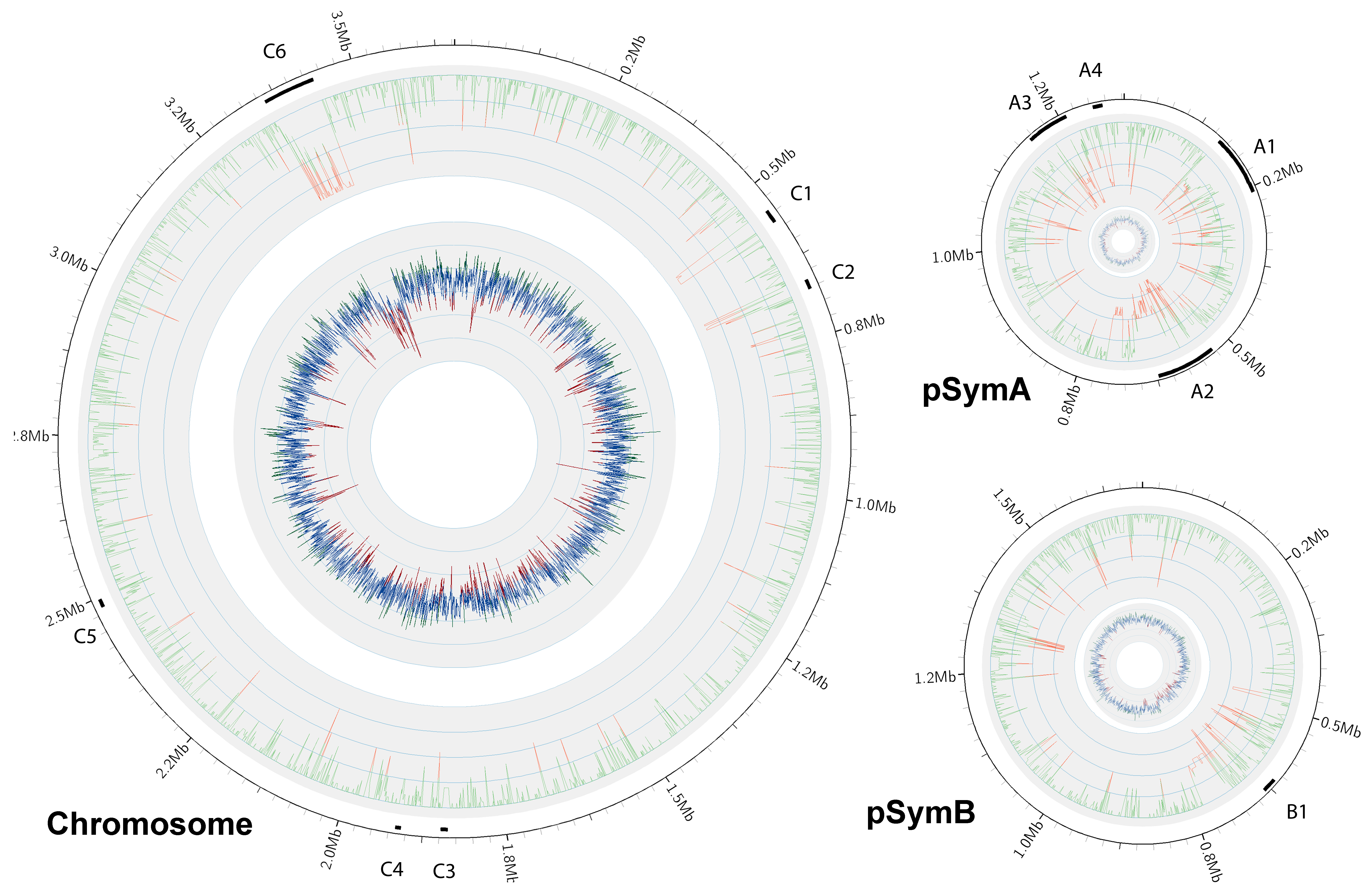

Supplement: Supplementary file 5 — Additional file 5: Comparative genomic analysis, comparing S. meliloti AK21 CGH data with data for other sequenced S. meliloti strains. Circular representation of the three main replicons of S. meliloti: chromosome, chromid pSymB and megaplasmid pSymA. The outer circle shows the genomic position, in Mb, according to the Rm1021 genome annotation. The next four concentric circles represent the missing genes in the analyzed S. meliloti genomes. The number of genomes that have lost the indicated genes is shown from the outer part of the diagram to the inner part, with five genomes in the first circle and 20 genomes in the last circle. Finally, the innermost group of circles indicate the GC skew determined for Rm1021. Highlighted regions in the outer circle correspond to the missing regions described in the main text and detailed in Table 1. The lower G + C content of Rm1021 corresponds to the regions missing from most of the S. meliloti genomes. The in silico comparison was carried out with BLAST + software and by BBH-based analysis (Camacho et al. 2009; Altenhoff and Dessimoz 2009). We created local protein databases for each genome, updated in May 2013, and established pairwise comparisons with Rm1021: Rm1021 [NCBI:PRJNA57603, PRJNA19]; SM11 [NCBI:PRJNA159685, PRJNA41117]; AK83 [NCBI:PRJNA52607, PRJNA41993]; 2011 [NCBI:PRJNA193772, PRJNA187276]; BL225C [NCBI:PRJNA52605, PRJNA42477]; GR4 [NCBI:PRJNA184823, PRJNA175860]; Rm41 [NCBI:PRJEA176372, PRJEB436]; 1A42 [NCBI:PRJNA199493, PRJNA167584]; 4H41 [NCBI:PRJNA199075, PRJNA169747]; 5A14 [NCBI:PRJNA199492, PRJNA167593]; A0641 M [NCBI:PRJNA199490, PRJNA167594]; A0643DD [NCBI:PRJNA199488, PRJNA167595]; AE608H [NCBI:PRJNA199486, PRJNA167596]; AK11 [NCBI:PRJNA199484, PRJNA167597]; AK75 [NCBI:PRJNA199482, PRJNA167598]; C0431A [NCBI:PRJNA199480, PRJNA167599]; C0438LL [NCBI:PRJNA199478, PRJNA167600]; CCNWSX0020 [NCBI:PRJNA180010, PRJNA75085]; and, H1 [NCBI:PRJNA199476, PRJNA167601]. A defined protein was considered to be lost if [file 40064_2015_1062_MOESM5_ESM.tiff]
